# Collateral benefits of restricted insecticide application for control of African trypanosomiasis on *Theileria parva* in cattle: a randomized controlled trial

**DOI:** 10.1186/1756-3305-7-432

**Published:** 2014-09-08

**Authors:** Dennis Muhanguzi, Kim Picozzi, Jan Hatendorf, Michael Thrusfield, Susan Christina Welburn, John David Kabasa, Charles Waiswa

**Affiliations:** Department of Biomolecular and Biolaboratory Sciences, College of Veterinary Medicine Animal Resources and Biosecurity, Makerere University, P.O. Box 7062, Kampala, Uganda; Division of pathway Medicine, Centre for Infectious Diseases, School of Biomedical Sciences, College of Medicine and Veterinary Medicine, The University of Edinburgh, Chancellor’s Building, 49 Little France Crescent, Edinburgh, EH16 4SB UK; Department of Public Health and Epidemiology, Swiss Tropical Institute, Socinstrasse 57, Basel, CH-4002 Switzerland; University of Basel, Petersplatz 1, Basel, 4003 Switzerland; Royal (Dick) School of Veterinary Studies, The University of Edinburgh, Edinburgh, EH25 9RG UK

**Keywords:** Collateral benefits, Endemic stability, East coast fever, p104-based PCR, Restricted application protocol (RAP), *T.parva*, Tororo district

## Abstract

**Background:**

Tick and tsetse-borne diseases (TTBDs) constrain livestock production in tropical and subtropical regions of the world. Of this community of endemic diseases, East coast fever (*T.parva*) is the most important tick-borne disease (TBD) accounting for 70% of all losses due to TBDS in this region where control efforts target either tsetse or TBDs and seldom both. In those instances where simultaneous pyrethroid insecticide TTBD control is implemented, collateral benefits of tsetse control on TBD control have not been quantified. In the interest of guiding future TTBD control efforts, the effect of restricting pyrethroid insecticides to the legs, belly and ears (RAP) of cattle for tsetse and trypanosomiasis control on *T.parva* prevalence in crop-livestock production systems in Tororo district, south-eastern Uganda was determined.

**Methods:**

We randomly allocated 16 villages to diminazene diaceturate (DA) and 3 graded RAP (25%, 50% and 75% of village herd sprayed respectively) treatment regimens. All cattle were ear-tagged, treated with diminazene diaceturate (DA) and those in regimens 2-4 received monthly graded RAP. Blood samples taken fourteen days post DA treatment and once three monthly were analysed by molecular techniques for *T.parva*.

**Results:**

In total, 8,975 samples from 3,084 animals were analysed. Prevalence of *T.parva* varied between 1-3% in different treatment regimens. RAP regimens were associated with slightly lower average risk of infection compared to DA. However, the confidence interval was broad and the result was not statistically significant. There was no evidence of a dose response relationship between graded RAP and *T.parva* prevalence. These findings are discussed herein with regard to endemic stability development to different TBDs.

**Conclusions:**

We found only a slight effect of RAP on *T.parva* infection. Since sample size determination was based on trypanosomes incidence, the study was underpowered given the low *T.parva* prevalence. While the findings need to be confirmed in future studies, the observed slight reduction in the risk of infection with *T.parva* might not compromise endemic stability*.*

## Background

Endemic hemoparasitic diseases of livestock mainly TTBDs constrain livestock production in tropical and subtropical regions of the world [1–3]. Of this community of endemic vector-borne diseases; East coast fever (ECF) caused by *Theileria parva* is the single most important TBD in the East African region costing about 70% of all losses due to TBDs [4–8]. Zoonotic trypanosomiasis with a Reservoir in cattle population negatively impacts on human health [9]. The cost of TTBDs to the livestock sector is in form of mortality, morbidity, treatment or control [4, 6]. However, the importance of each of these diseases cannot be singled out accurately as they co-exist in the same livestock populations [5]. In south-eastern Uganda, for example, TTBDs constrain livestock populations and compound poverty levels, with about 34% of all livestock holders subsisting on less than 1.24 US$ per day [10–12]. In Sub-Saharan Africa, veterinary services have largely been privatised and decentralised [13–15] resulting in small holder farmers with inelastic budgets being largely in charge of disease control programs [14–16]. To be beneficial and sustainable there is a need for use of integrated livestock disease control methods that are cheap and targeting more than one endemic livestock disease. Livestock disease control managers should therefore promote integrated low-cost and environmentally sustainable technologies for TTBD control [16, 17].

A recent study in south-eastern Uganda, for example, reported 62% of all farmers using amidines (Amitix® and Noratraz®) for TBD control, which have no significant insecticidal activity to tsetse flies [18]. Similarly, donor and government-led livestock disease control programs in this area don’t always plan simultaneous TTBD control. However, these areas are endemic for AAT, acute HAT and TBDs. In those tsetse control programmes designed to benefit tick control, like in the stamp out sleeping sickness (SOS) program in Uganda [19, 20], the collateral benefits of using pyrethroid insecticides for tsetse and trypanosomiasis control on TBDs have, to our knowledge, not been evaluated. In addition, the effect of simultaneous TTBD control by use of pyrethroid insecticides by about 38% of the farmers in this region has not been assessed. As such, benefits of simultaneous TTBD control by use of pyrethroid insecticides have generally been evaluated only in a few instances [21].

In order to prevent the merger of the chronic and acute forms of HAT triggered by the northerly spread of acute HAT caused by cattle restocking in south-eastern Uganda, over 0.5 million cattle were sprayed by restricting pyrethroid insecticides to the legs and bellies of cattle [20, 22]. This method otherwise called restricted application of pyrethroid insecticides (RAP) has previously been proved to be effective against tsetse and trypanosomiasis [17]. As such the SOS program used this technology to effectively control the merger of the two forms of sleeping sickness triggered by cattle restocking in the north and south-eastern Uganda following about 20 years of insurgence [19, 20]. The said insurgence and the need to have people in concentration camps led to loss of a large population of cattle over the 20 year period. When people were resettled in their former villages after the insurgence, the government provided them with livestock (mainly cattle), which was sourced from far south in the Busoga region which is known to be endemic for *T.b rhodesiense*. As a result, there was introduction of previously non-endemic *T.b rhodesiense* into south-eastern Uganda especially Teso region. This caused a northerly spread of *T.b rhodesiense* facilitated by unregulated cattle trade and movement in districts north of the Teso region.

In contrast to whole-body treatment, RAP has previously been reported as unlikely to disrupt endemic stability to TBDs [13, 17]. This is thought to be as a result of RAP maintaining a dampened exposure to tick-borne pathogens as calves and development of solid immunity against clinical disease as adults [23, 24]. In crop-livestock production systems where cattle are continuously exposed to ticks [25, 26], endemic stability is beneficial to reducing losses due to TBDs. However, there is no much information about the collateral benefit of using pyrethroid insecticides for tsetse and trypanosomiasis control on tick-borne hemoparasites populations. This study was carried out to quantify the effect of applying RAP, which is primarily used to control tsetse flies, on *T.parva* prevalence. We selected *T.parva* among other tick-borne hemoparasites because it is the causative agent of ECF, which accounts for up to 70% of all livestock sector losses in the region [4, 7, 27]. We further aimed at establishing if there was any dose response relationship, by spraying varying proportion of the village cattle herd. This information will guide control managers and policy makers in planning and evaluating simultaneous TTBD control programs and to leverage resource allocation to human and livestock vector-borne disease control programs.

## Methods

### Study area; study village selection and allocation to treatment regimens

This study was carried out in Tororo District, south-eastern Uganda between June 2012-December 2013. The location, livestock production systems, climate and vegetation of Tororo District have been described elsewhere [25, 28]. Cattle herds of 16 villages were randomly allocated to one of 4 different treatment regimens namely; 1) two treatments with diminazene diaceturate (DA) administered at a dose of 0.01 g/kg live body weight (bwt) by deep intramuscular injections at 40-day intervals (DA); 2) DA and RAP of 25% of village herds; 3) DA and RAP of 50% of village herds and 4) DA and RAP of 75% of village herds. Each of the regimens was trialed in 4 villages. We screened 57 villages for eligibility and collected data on basic socio-demographics, trypanosome and *T.parva* [28] prevalence with molecular techniques. Twenty-seven villages fulfilled the eligibility criteria of i) a cattle population of > =50 and ii) a trypanosome prevalence of > =15%. In order to randomly select 16 villages, we generated 100 unique allocation sequences, which fulfilled the condition of a minimum distance of 2 km between neighbouring villages. This was to minimize contamination effects from different intervention arms. Finally, one allocation sequence was selected randomly.

### Description of field cattle treatments

All cattle in the selected villages were ear tagged and treated with a long acting diminazene diaceturate (DA) containing cyanocobalamin (vitamin B12) and hydroxocobalamin (Vitamin B12a) (Veriben B12®; Ceva santé animale, France), twice forty days apart at the beginning of the trial. Veriben B12® was administered at a dose of 0.01 g/kg live body weight (bwt) by deep intramuscular injections to rid cattle of trypanosome infections. Livestock-keepers, their household particulars (village, parish, county) and cattle demographics (age, sex, breed,) were entered on a herd structure register at the time of introduction into the intervention which was updated once three monthly. In regimens 2-4; different proportions (25%, 50% and 75%) of the village cattle herd were sprayed once every 28 days in what is referred to here as graded RAP. An emulsifiable deltamethrin concentrate (Vectocid®, Ceva Interchem, Tunis) spray was applied in the recommended concentration of 1: 1000 (Vectocid to water parts) on legs, belly and ears as previously described [17] for control of tsetse and ticks. Cattle in regimen 1 only received two doses of DA forty days apart at the beginning of the trial. Blood samples were taken 14 days post the last Veriben B12® injection and repeated once three monthly for 18 months of the trial. All cattle in the non-RAP villages were administered with Veriben B12® injections at the end of the trial since they were at a higher risk of infection with different trypanosome species.

### Cattle blood sample collection

About 125 μl of blood were collected from the middle ear vein and applied onto the classic Whatman FTA® cards (Whatman Bioscience, Cambridge, UK) avoiding cross contamination of the four samples on each card [29, 30]. The samples were then allowed to air-dry, labelled with cattle tag number, treatment regimen, sampling number, village name, parish, sub County, County and date of collection. They were packed in foil pouches with a silica gel desiccant (Sigma Aldrich, Co., Life sciences, USA) prior to shipping to the University of Edinburgh, UK for analysis.

### DNA extraction

DNA was extracted and eluted in Chelex®100 resin (Sigma Aldrich, Co., Life sciences, USA) from 3 mm FTA test sample or empty negative control discs according to a previously described protocol [30, 31]. Eluted DNA samples were kept at -20°C for long-term PCR analyses or 4°C if they were to be analysed within a few days after extraction. The Sources, quality and storage of DNA used as positive control DNA for *T.parva* p104-based PCR [32] have been recently described [28].

### *T.parva*detection by kDa antigen (p104) based PCR

Eluted DNA samples were screened for *T.parva* using a single pair of primers (IL4243; 5-GGC CAA GGT CTC CTT CAG AAT ACG-3 and IL3232; 5-TGG GTG TGT TTC CTC GTC ATC TGC-3) derived from p104 single copy gene [32]. This primer set amplifies a 277 bp fragment of a highly conserved segment of p104 gene making it a very specific and sensitive target for *T.parva* diagnosis [32, 33]. The sensitivity and the choice for p104-based PCR for *T.parva* detection have recently been discussed in our previous study [28]. PCR was performed in a 25 μl reaction volume; 20 μl of which was the PCR master mix containing 2.55 μl of 10 x-reaction buffer (670 mM Tris-HCl pH 8.8, 166 μM (NH4)_2_SO_4_, 4.5% Triton X-100, 2 mg/ml gelatin) (Fisher Biotech), ImM MgCl_2_, 200 μM of each dNTP, 5 μM each of the IL3232 and IL4234 primers, 0.7 U of BioTaq DNA polymerase (Fisher Biotech), 14.55 μl RNase-free water and 5 μl of sample DNA or positive control DNA or negative control eluate [32, 33]. PCR was carried out in a DNA Engine Dyad® Cycler (PTC-0221, Bio-Rad Laboratories Inc.) at cycling conditions including a denaturation step at 95°C for 5 minutes, 30 cycles of denaturation at 94°C for 30 s each cycle, annealing at 65°C for 30 s, extension at 72°C for 1 minute, final elongation step at 72°C for 5 minutes [32, 33]. PCR products were electrophoresed in 1.5% agarose (Bio Tolls Inc. Japan), stained in GelRed™ (Biotium, Inc., USA) and visualised on an ultraviolet transilluminator.

### Statistical analyses

The primary analysis investigated the impact of RAP on the incidence risk ratios of *T.parva* infection using generalized linear mixed models with a Poisson distribution and a logarithmic link function. To account for correlation within clusters, villages were included as gamma distributed random effects. The logarithm of time under observation, i.e. the interval between the first and last time an individual animal was sampled and included as offset variable. To assess the intervention effect over time, prevalences after 6, 12 and 18 months of follow up were compared using mixed models with binary outcome and logit link function. The original idea of modelling the proportion of animals treated with RAP as a dose response relationship was abandoned because we did not observe decreasing infection risk with increasing proportion of treated animals. Therefore, the results for the different treatment regimens compared to the control regimen are presented. All statistical analyses were performed using the R statistical software v 3.0.2 except the Poisson random effect models, which were performed in STATA v 12.1.

### Cattle biophysical monitoring data entry and analysis plan

**Figure 1:**
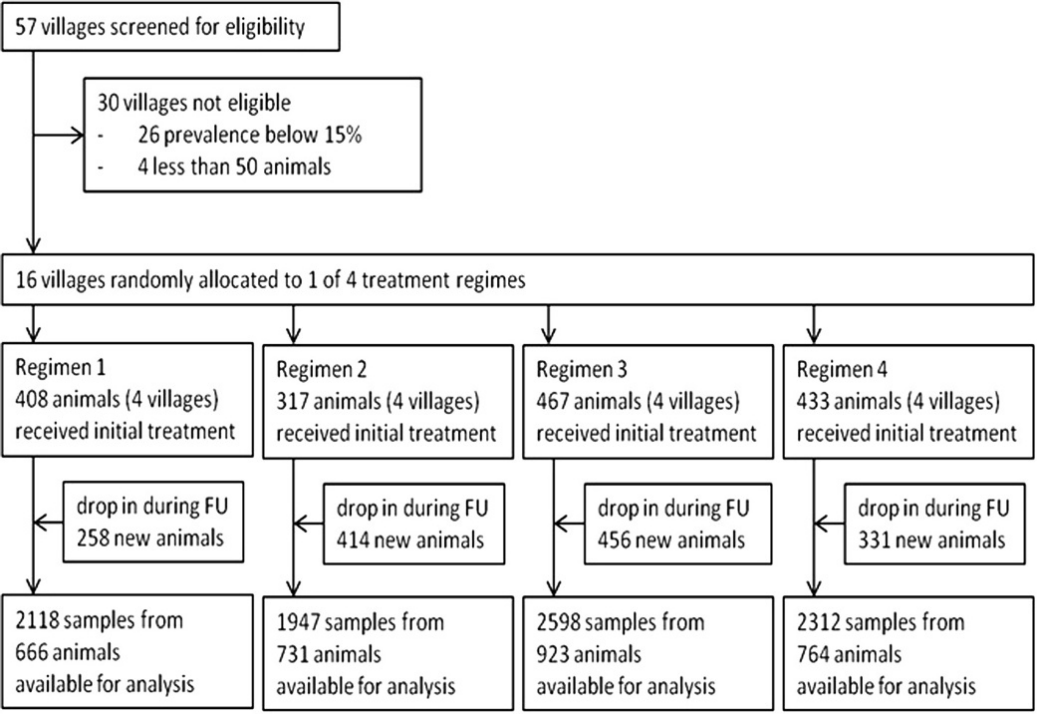
**Study flow.** Regimen 1: Diminazene diaceturate injections (DA); (0.01 g/kg body weight) forty days apart at the beginning of the trial. Regimen 2: DA and 25% RAP. Regimen 3: DA and 50% RAP. Regimen 4: DA and 75% RAP. Median time of follow up-FU (time difference between first and last sampling of individual animals) was 12 months in each of the 4 treatment groups.

Eight thousand nine hundred and seventy five (8,975) blood samples were collected from 3,084 cattle, 1,625 of which were introduced at the beginning of the trial and 1,459 were introduced during follow-up. Samples were taken at seven sampling points; 14 days post last DA treatment and there after once every three months for 18 months. The number of blood samples collected in each of the four regimens, data entry and analysis plan are summarised in Figure 1.

### Ethical clearance

This study was reviewed by the Makerere University College of Veterinary Medicine Animal Resources and Biosecurity ethical review board for compliance to Animal use and Care standards. It was then forwarded to the Uganda National Council for Science and Technology and approved under approval number HS1336.

## Results

### Demographic characteristics 2 weeks post the second diminazene treatment

One thousand six hundred and twenty seven cattle were sampled 14 days post DA treatment in the 4 treatment regimens and examined to determine *T.parva* prevalence. Table 1 summarises the prevalence of *T.parva* in the 4 regimens before and after treatment and the study population demographic characteristics namely age, sex and breed.

**Table 1:** **Baseline characteristics of the study population (2 weeks after initial treatment)**

Prevalence	Treatment groups			
	1	2	3	4
**A) Infection status 9 months before treatment and 2 weeks after initial treatment**				
Number sampled (n)	408	320	467	432
n villages	4	4	4	4
Prevalence before treatment [%]*	7.5	4.4	2.4	8.3
Prevalence after treatment [%]	1.0	1.6	1.7	2.1
**B) Demographic characteristics**				
**Population attributes**	**n (% Within group)**			
Sex [n (%)]				
Male	175 (43%)	109 (34%)	165 (35%)	176 (41%)
Female	192 (47%)	193 (60%)	269 (58%)	226 (52%)
Castrate	41 (10%)	18 (6%)	33 (7%)	30 (7%)
Breed [n (%)]				
Boran × African short horn Zebu (Nkedi)	394 (97%)	296 (92%)	450 (96%)	423 (98%)
Boran × Holstein Friesian	8 (2%)	23 (7%)	0 (0%)	3 (1%)
African short horn Zebu (Nkedi)	6 (1%)	1 (0%)	17 (4%)	6 (1%)
Age in years [n (%)]				
0.0-1.0	48 (12%)	40 (12%)	35 (7%)	72 (17%)
1.1-3.0	188 (46%)	153 (48%)	188 (40%)	150 (35%)
3.1+	172 (42%)	127 (40%)	244 (52%)	210 (49%)

*determined 12 months before treatment (n = 321, 430, 572, 576).

### Prevalence of *T.parva*by treatment regimen and time

**Figure 2:**
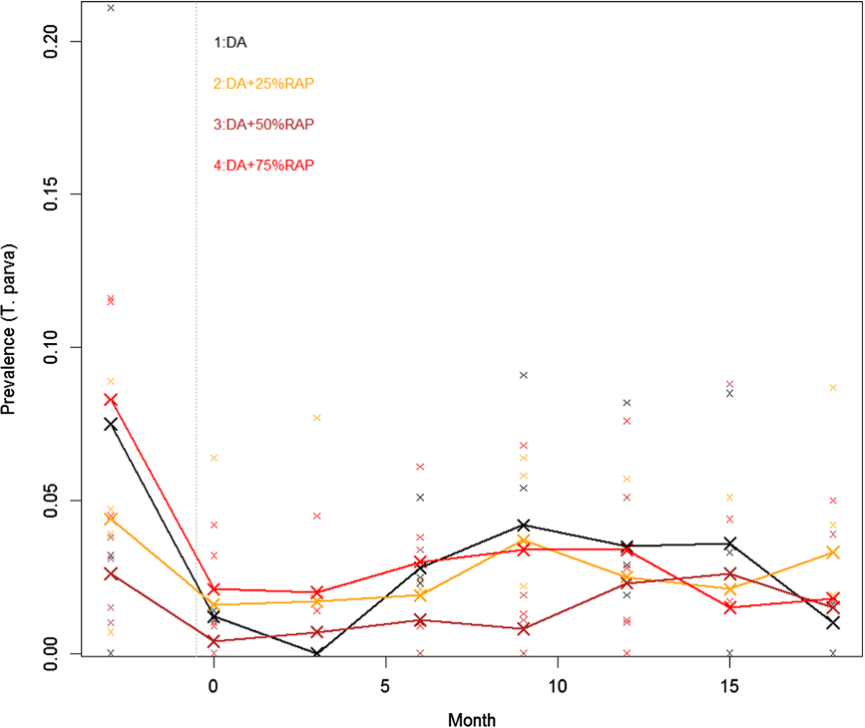
***T.parva*** **prevalence by time in different regimens.** Lines represent prevalences calculated as unweighted mean of the 4 village prevalences.

The prevalence of *T.parva* about 9 months before initial treatment in different regimens varied greatly from 2.4-8.3%. At 14 days post DA injections (denoted as time 0), *T.parva* prevalence dropped to 1.5% with the rate of fall proportional to the baseline prevalence in all regimens. Thereafter*, T.parva* prevalence slightly increased in all regimens up to month 9 of the trial but prevalence remained below 5%. In regimen 1, *T.parva* prevalence continued to decline 3 months post DA administration up to 3 months of the trial and there after increased up to 12 months of the trial. In all regimens there was a general decrease in *T.parva* prevalences from 12 months of the trial corresponding to the reduction in the number of animals in the trial. The prevalence of *T.parva* only reached pre-intervention levels in regimen 3 at month 9 up to month 15. In the rest of the regimens including DA regimen the prevalence of *T.parva* fluctuated at levels below the pre-intervention levels (Figure 2).

### *T.parva*prevalences in different treatment regimens over time

Drop-in effect (cattle introductions) during follow-up did not have any effect on *T.parva* prevalence up until month 9 of the trial where dilution effect of about 0.01 units on the infection levels was observed mainly in regimens 1 and 4. Drop-in effect had a particularly high dilution effect of 0.04 units on *T.parva* prevalence in regimen 2 at end of the trial (Month 18). Table 2 summarises *T.parva* prevalence in each regimen over time with and with -out drop-in effects at each sampling time.

**Table 2:** **Overall prevalence of** ***T.parva*** **in different treatment regimens over an 18 months follow-up period**

Regimen	Month	n	***T.parva***	n*	T.parva*
1	0	408	0.01	408	0.01
2		317	0.02	317	0.02
3		467	0.00	467	0.00
4		433	0.02	433	0.02
1	3	273	0.00	268	0.00
2		294	0.02	294	0.02
3		415	0.01	414	0.01
4		355	0.02	338	0.02
1	6	359	0.03	316	0.03
2		308	0.02	264	0.02
3		375	0.01	326	0.01
4		361	0.03	309	0.04
1	9	307	0.04	257	0.05
2		299	0.04	231	0.04
3		383	0.01	259	0.01
4		320	0.03	226	0.04
1	12	404	0.04	331	0.04
2		285	0.03	186	0.03
3		426	0.02	297	0.02
4		353	0.03	230	0.04
1	15	169	0.04	150	0.03
2		234	0.02	124	0.02
3		270	0.03	148	0.01
4		264	0.02	136	0.03
1	18	198	0.01	173	0.01
2		210	0.03	90	0.07
3		262	0.02	117	0.01
4		226	0.02	128	0.02

*Only animals that received baseline treatment are included.

### Six monthly incidences and ***T.parva***point prevalence in RAP and non-RAP regimens

The proportion of positive samples was 2.1% slightly higher in non-RAP compared to 1.8% in the RAP regimens but the difference was not statistically significant (Incidence Risk Ratio (IRR)=0.6; 95%CI; 0.22-1.67; P=0.65) as summarised in Table 3. Similarly, the risk of infection with *T.parva* was slightly higher in non-RAP than RAP villages at 6 and 12 months of the trial but this difference was not significant (OR = 0.7 at each sampling time). The risk of infection was higher in RAP than non-RAP villages by 18 months of the trial (OR, 2.7; 95% CI; 0.37-12.73) as summarised in Table 3.

**Table 3:** ***T.parva*** **incidence during 18 months of follow-up**

Category	Animals (n)	Samples (n)	Episodes (n)	Positive (%)	IRR	95% CI	P
**A) Overall incidences**							
No RAP	666	2118	45	2.1	Ref		
RAP	2418	6857	121	1.8	0.80	0.30-2.14	0.65
**B) Prevalences at month 6, 12 and 18**							
	Animals; n (%)	Positive; n (%)	Crude OR	95% CI			
**i) Prevalence at month 6**							
No RAP	359	10 (3%)	Ref				
RAP	1044	21 (2%)	0.70	0.27-1.8			
**ii) Prevalence at month 12**							
No RAP	404	14 (3%)	Ref				
RAP	1064	29 (3%)	0.67	0.24-1.91			
**iii) Prevalence at month 18**							
No RAP	198	2 (1%)	Ref				
RAP	698	15 (2%)	2.17	0.37-12.73			

### Spatial effect on *T.parva*prevalence in different regimens over time

**Figure 3:**
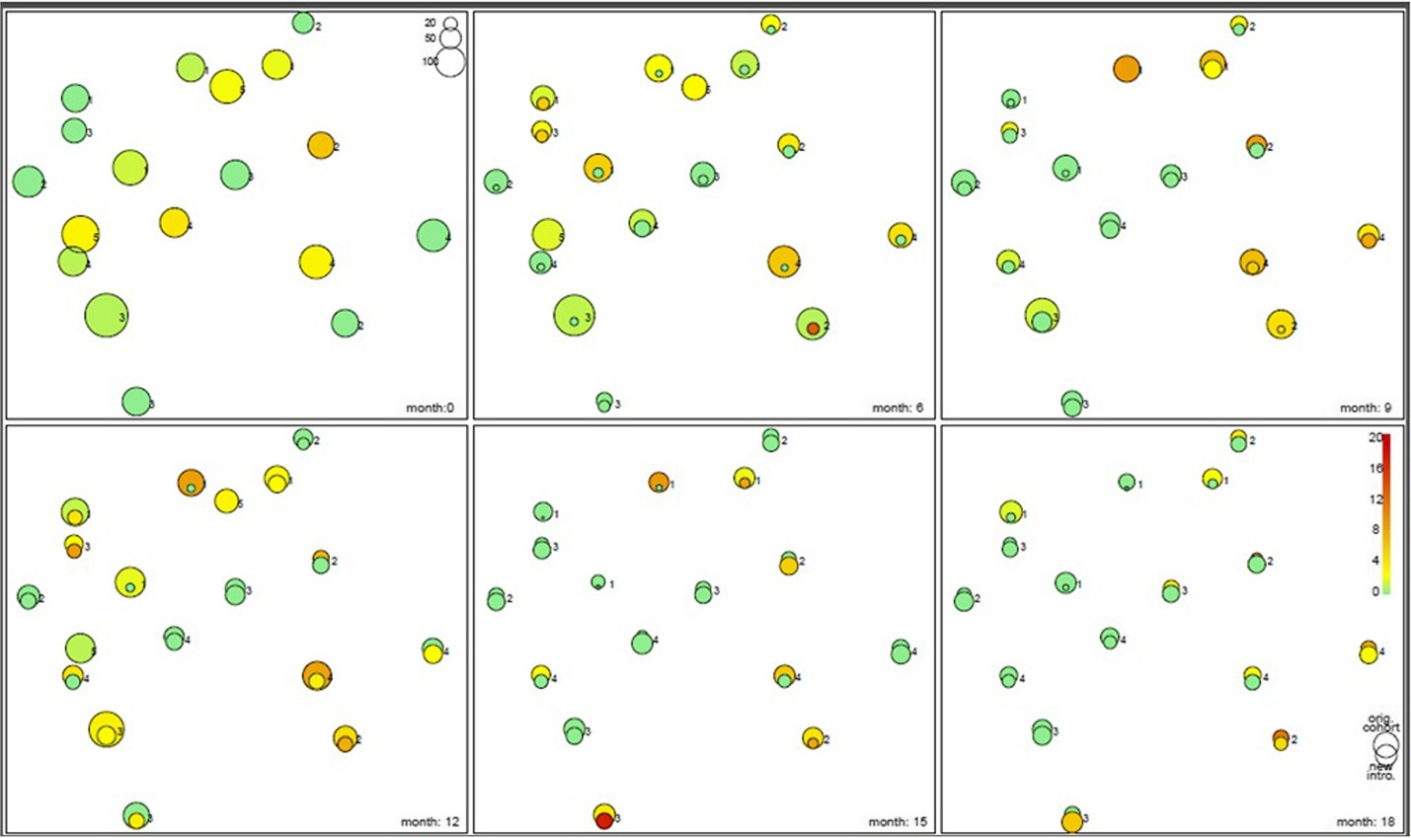
**Spatial distribution of** ***T.parva*** **over an 18 months period.** The colour represents the prevalences. The circle area is proportional to the number of animals sampled.

*T.parva* prevalence was highly variable at each of the seven sampling points in the four treatment groups (Table 2). Moderate to high (5-10%) *T.parva* prevalences were randomly distributed in the north, north-eastern and the south-eastern parts of the district regardless of treatment regimens (Figure 3).

## Discussion

A randomized controlled trial was carried out in Tororo District to determine the effect of applying RAP for tsetse control on *T.parva* prevalence and how it would change (if at all) with increasing RAP herd coverage. One thousand six hundred and twenty five cattle were introduced into one Non-RAP regimen and three graded RAP (25%, 50% and 75% herd coverage) regimens. About the same number of cattle (1,459) was introduced in the four regimens during the 18 months of follow-up.

At the beginning of the trial, 47% of all cattle were females while 46% of all cattle were above 3 years of age. Such population structure of retaining more old cattle with a female to male (whole and neutered males) ratio of nearly 1 has previously been seen as a drive to creating a mass of draught power animals [28, 34]. This implies that improving livestock health by controlling TTBDs will help farmers use draught power, cattle manure and integrate crop and livestock production thereby reducing poverty and hunger [2, 13, 35–37].

*T.parva* prevalence varied greatly in different regimens before (2.4-8.3%) the intervention. Medium to high (4.0-7.0%) *T.parva* prevalence was observed persistently in villages in the north, north-eastern and the south-eastern parts of the district (Figure 3) regardless of the treatment regimen. This was previously associated with the differences in tick challenge between different intervention villages with *T.parva* prevalence proportional to the level of tick challenge in each intervention village [28, 38]. RAP herd coverage was not associated with a proportionate decrease in *T.parva* prevalence probably as a result of village level effect of differences in tick abundance and *T.parva* transmission [28, 38].

Other village level effects could have contributed to this dose- response relationship distortion albeit in unknown proportions. These factors include; differences in residual infections post DA injections, farming practices including differences in veterinary care between villages and drop-in effects; to a very little extent after 9 months into the trial.

There was a drastic decline in *T.parva* prevalence 14 days post DA treatment much as DA is not specifically and routinely used in the treatment of *T.parva* infections. The reason for this decline in the *T.parva* prevalence in cattle post DA treatment could be 2 fold.

DA has previously been known to have some action on members of the class Piroplasmida (*Babesia, Theileria*, and *Cytauxzoon*) in addition to the primary targets for this drug; trypanosomes [39]. In areas like south-eastern Uganda where TTBDs are endemic, mixed infections with *Babesia, Anaplasma, Theileria* and trypanosomes are common. These infections are usually associated with marked anaemia and lethargy [40]. Veriben B12® contains vitamins B12 and B12a which alleviate anaemia in addition to having specific effect on trypanosomes, *Anaplasma* and *Babesia* species. Therefore, the second plausible explanation of the decline in the pre-intervention *T.parva* prevalence 2 weeks post Veriben B12® treatment is self-cure following alleviation of anaemia and cleaning animals of concurrent tsetse and other tick-borne infections.

The risk of infection and therefore the prevalence of *T.parva* was lower in RAP regimens (2-4) than the DA treatment regime. However, the incidence rate ratios associated with RAP and non RAP regimens were not statistically significant. Due to the generally low prevalence of *T.parva* the study was underpowered, since sample size determination was based on trypanosomiasis infection, which occurred at higher prevalences of about 30% [41]. The prevalence of *T.parva* was only higher in RAP regimens than regimen 1 only at month 18 of the trial. This is likely to have been caused by the decline in animal population and drop-in effect (cattle introduction) or simply by stochastic effects since the overall number of positive animals was 18. Previously, it had been suggested that restricting pyrethroid insecticides/acaracides was likely to reduce tick populations and maintain a small force of infection with tick-borne hemoparasites in cattle [17, 42]. We found only a slight effect of RAP on *T.parva* infection, which is in line with this school of thought. Since sample size determination was based on trypanosome incidence, the study was underpowered given the low *T.parva* prevalence. While the findings need to be confirmed in future studies, the observed reduction in the risk of infection with *T.parva* might not compromise the endemic stability as had been previously suggested.

Restricted application of pyrethroid insecticides was applied once every 28 days; an application regime that is longer than what is recommended for three host tick *R. appendiculatus* but sufficient for tsetse control [13]. This could have maintained a small population of ticks on cattle there by maintaining a small prevalence of *T.parva* in all regimens across over the follow-up time. This in itself varied greatly in different villages as has been explained depending on the variation in tick-abundance in different villages as previously observed [28, 38]. This reaffirms that use of RAP is likely to maintain endemic stability in an epidemiological situation that is beneficial in small holder crop-livestock production systems like in south-eastern Uganda where cattle are constantly exposed to ticks and therefore tick-borne infections [25, 26]. This, together with the fact that RAP is environmentally benign have been reported as some of the collateral benefits of this technology [17, 43]. RAP uses about 20% of total amount of pyrethroid insecticides compared to the amounts needed for whole body spray [17, 44]. This minimises damage to the invertebrate dung fauna, which break down dung and add manure to soils in crop-livestock production systems [45, 46]. Our findings that RAP had a slight effect on *T.parva* infection indicating that it might not compromise endemic stability to *T.parva* further proves that RAP is a promising future farmer- based technique for simultaneous TTBD control after more field based evaluations [17, 42].

However, this should be extended to the possibility of RAP maintaining endemic stability to other TBDs with care because the ease with which endemic stability is likely to develop to each TBD is different [13]. The probability of clinical disease development following infection with different tick-borne hemoparasites, for example, varies greatly with age [47–49]. Whereas less severe disease is likely to develop in calves below 6 months of age in anaplasmosis, babesiosis and cowdriosis as a result of maternal immunity in this age group, this relationship is less pronounced in *T.parva* infections [13, 50]. However, this phenomenon is very important in ECF epidemiology. Further studies are therefore recommended to investigate the effect of RAP for tsetse and trypanosomiasis control on seroconversion and/or progression to different tick-borne clinical diseases. This will further broaden our understanding of how RAP is likely to affect endemic stability to different TBDs in different livestock production systems under different tick challenge levels.

## Conclusions

We found only a slight effect of RAP on *T.parva* infection. Since sample size determination was based on trypanosomes incidence, the study was underpowered given the low *T.parva* prevalence. While the findings need to be confirmed in future studies, the observed reduction in the risk of infection with *T.parva* might not compromise endemic stability to *T.parva* infections. However, this should be extended to the possibility of endemic stability development to other TBDs reservedly since the ease with which endemic stability develops to each TBD varies greatly. We therefore recommend that future studies to investigate the effect of RAP on sero-conversion and/ or progression to clinical disease due to different tick-borne hemoparasites be carried out in different livestock productions systems. This research discourse is recommended because endemic stability is important in reducing losses to TBDs in production systems where cattle are continuously exposed to ticks with very high force of infection of tick-borne hemoparasites.
